# Clinical indicators of adrenal insufficiency following discontinuation of oral glucocorticoid therapy: A Danish population-based self-controlled case series analysis

**DOI:** 10.1371/journal.pone.0212259

**Published:** 2019-02-19

**Authors:** Kristina Laugesen, Irene Petersen, Henrik Toft Sørensen, Jens Otto Lunde Jørgensen

**Affiliations:** 1 Department of Clinical Epidemiology, Aarhus University Hospital, Aarhus, Denmark; 2 Department of Primary Care and Population Health, University College London, London, United Kingdom; 3 Department of Endocrinology and Internal Medicine, Aarhus University Hospital, Aarhus, Denmark; Weill Cornell Medical College Qatar, QATAR

## Abstract

**Background:**

Biochemical adrenal insufficiency induced by glucocorticoid treatment is prevalent, but data on the clinical implications are sparse. We investigated clinical consequences of glucocorticoid-induced adrenal insufficiency after oral glucocorticoid cessation.

**Methods:**

We conducted a Danish population-based self-controlled case series utilizing medical registries. In this design each individual serves as their own control allowing event rates to be compared as a function of time and treatment. Clinical indicators of adrenal insufficiency were defined as diagnoses of gastrointestinal symptoms, hypotension, cardiovascular collapse, syncope, hyponatremia, and hypoglycaemia. We included 286,680 persons who discontinued long-term (≥ 3 months) oral glucocorticoid treatment. We defined five risk periods and a reference period (before treatment): period 0 (on treatment), withdrawal period (1 month before and after cessation), followed by three consecutive 2 month-risk periods after withdrawal (periods 2–4).

**Results:**

Median age at cessation was 69 years and 57% were female. Median treatment duration was 297 days and median cumulative dose was 3000 mg prednisolone equivalents. The incidence rates of hypotension, gastrointestinal symptoms, hypoglycemia and hyponatremia were increased in the withdrawal period compared to before treatment started (reference period). Incidence rate ratios comparing the withdrawal period with the reference period were 2.5 [95% confidence interval (CI): 1.4–4.3] for hypotension, 1.7 (95% CI: 1.6–1.9) for gastrointestinal symptoms, 2.2 (95% CI: 0.7–7.3) for hypoglycemia, and 1.5 (95% CI: 1.1–2.0) for hyponatremia. During 7 months of follow up, the rates of hypotension and gastrointestinal symptoms remained elevated compared to the reference period. Risk factors included use of antibiotics, increasing average daily dose of glucocorticoids, cumulative dose, and age.

**Conclusion:**

Oral glucocorticoid withdrawal was associated with adverse outcomes attributable to adrenal insufficiency. Our study underscores the need for future research to establish evidence-based clinical guidance on management of patients who discontinue oral glucocorticoids.

## Introduction

Primary adrenal insufficiency and secondary adrenal insufficiency due to a pituitary disorder are rare but serious conditions necessitating appropriate replacement therapy [1–3]. In contrast, adrenal insufficiency induced by pharmacological glucocorticoid treatment, *i*.*e*., iatrogenic or tertiary adrenal insufficiency, is highly prevalent but the clinical implications are less certain [4, 5].

Cortisol regulates an array of vital physiologic functions related to maintenance of basal and stress-related homeostasis [6–8]. The cardiovascular response to stress depends on cortisol-mediated activation of adrenergic receptors [7]. Cortisol also acts in concert with glucagon, catecholamines, and growth hormone to stimulate hepatic glucose output and lipolysis [7], and exerts effects that dampen stress-induced inflammatory and immune responses [7].

Glucocorticoid treatment suppresses the hypothalamic-pituitary-adrenal (HPA) axis, which may compromise endogenous cortisol secretion in response to stress and thus induce a state of relative adrenal insufficiency [1, 2]. In addition, discontinuation of glucocorticoid treatment can induce prolonged or even permanent suppression of endogenous cortisol secretion, as assessed by biochemical stimulation tests [4, 9–15]. A recent meta-analysis estimated a 50% pooled risk of biochemical adrenal insufficiency among oral glucocorticoid users [4]. This is noteworthy, considering that the annual prevalence of systemic glucocorticoid use is 3% in the Danish population [16]. Nonetheless, current knowledge regarding the clinical implications of glucocorticoid-induced adrenal insufficiency is restricted to anecdotal reports of fatigue, muscle weakness, gastrointestinal symptoms, hypotension, syncope, cardiovascular collapse, hyponatremia, and hypoglycemia [17–19]. The knowledge gap is reflected in current guidelines by the lack of evidence-based recommendations about management of iatrogenic adrenal insufficiency [20, 21]. We therefore conducted a population-based self-controlled case series analysis of clinical indicators of adrenal insufficiency during and after withdrawal of oral glucocorticoids. We defined putative clinical indicators of adrenal insufficiency and reported incidence rates before and after withdrawal of glucocorticoid treatment. We then used a self-controlled case series design [22, 23], in which each individual served as their own control and adverse event rates during and after glucocorticoid treatment were compared to a reference period.

## Material and methods

### Setting

Denmark provides its entire population with tax-supported healthcare, guaranteeing access to primary and secondary care free-of-charge. A unique personal civil registration number is assigned to all Danish residents at birth or upon immigration, enabling accurate and unambiguous individual-level linkage of relevant registries [24].

### Study population

**Fig 1:**
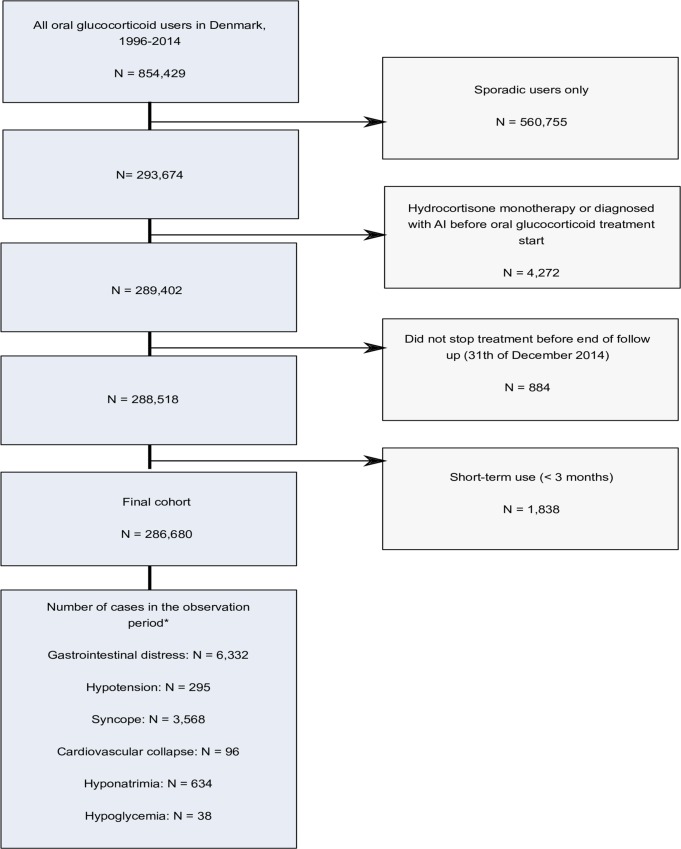
Flow chart of the study population. * The observation period ranges from 3 months before initiation of first long-term (≥ 3 months) oral glucocorticoid treatment to 7 months after the date of last glucocorticoid prescription (cessation).

We used the Danish National Prescription Registry [25] (DNPR) to identify all persons who discontinued long-term (≥ 3 months) treatment with oral glucocorticoids between January 1, 1996 and December 31, 2014 [26]. A flow chart of the study population is presented in Fig 1. The DNPR records information on pharmacy customers’ civil registration number, the redeemed medication’s classification code [Anatomical Therapeutic Chemical (ATC) classification system of the World Health Organization], date of dispensing, the number of packages dispensed, the number of tablets in a package, tablet strength, and amount dispensed, expressed in terms of “defined daily doses” (DDDs) developed by WHO. Subjects with a prior diagnosis of adrenal insufficiency, as well as subjects on continuous hydrocortisone treatment indicative of adrenal insufficiency, were excluded (n = 4,272). ATC codes for glucocorticoids and other relevant medications are provided in S1 Table.

### Exposure

**Fig 2:**
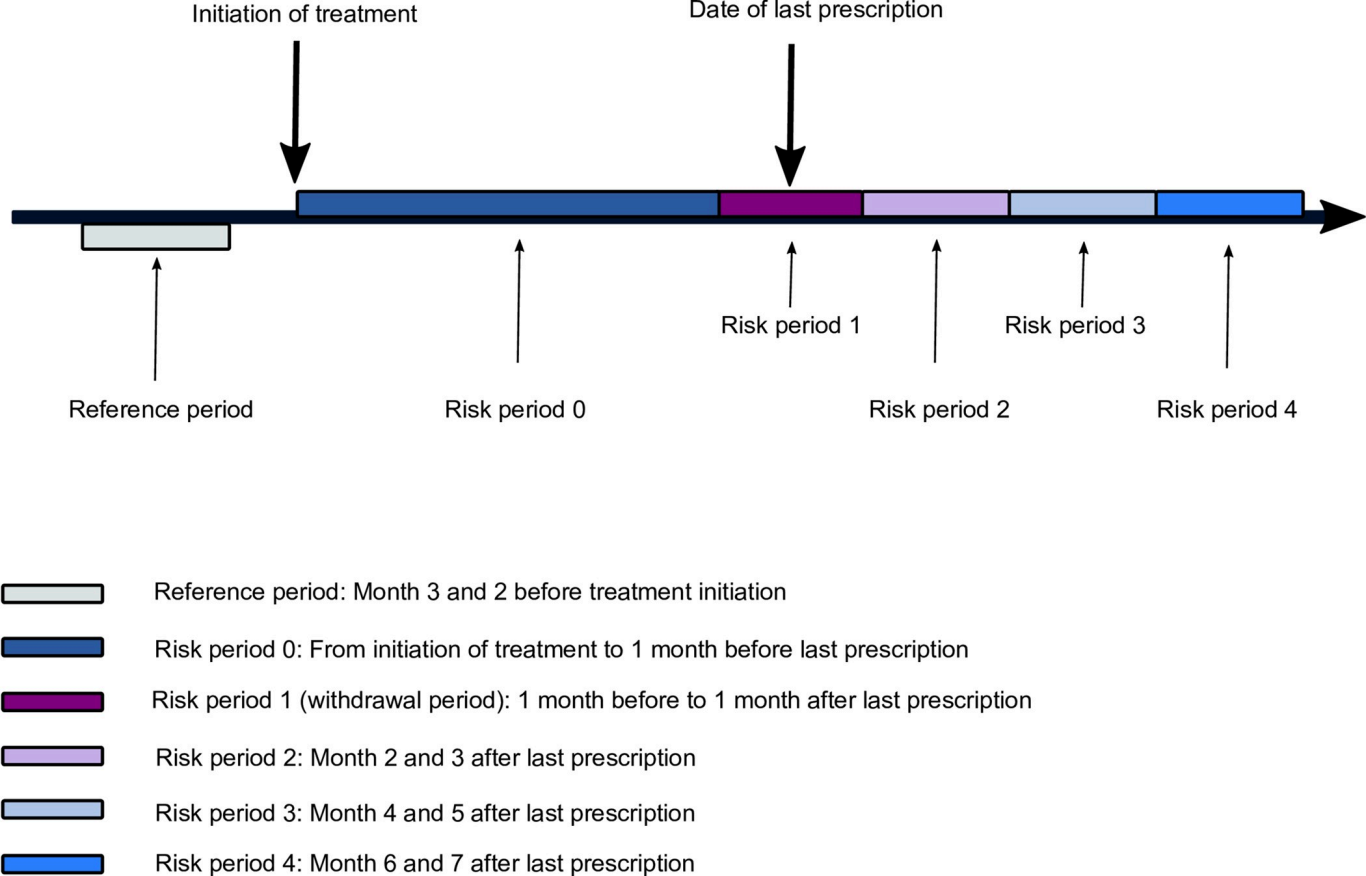
Observation period and defined risk periods for an individual person receiving oral glucocorticoid treatment who stops treatment before end of follow up.

We defined an observation period ranging from 3 months before initiation of an oral glucocorticoid to 7 months after the date of the last glucocorticoid prescription (cessation) for each person in the study population. The observation period was then divided into five risk periods (Fig 2). The definition of observation period and risk periods were based on findings of biochemical adrenal insufficiency in prior clinical studies [4, 5, 9–15]. Risk period 0 covered the time from the date of glucocorticoid therapy initiation to 1 month before redemption of the last glucocorticoid prescription. Risk period 1 (withdrawal period) covered the time from 1 month before redemption of the last prescription for a glucocorticoid to 1 month after this redemption (Days -30 to day 29). Risk period 2 covered months 2–3 after redemption of the last glucocorticoid prescription (Days 30 to 89); risk period 3 covered months 4–5 after redemption of the last glucocorticoid prescription (Days 90 to 149), and risk period 4 corresponded to months 6–7 after redemption of the last prescription (Days 150 to 210). Finally, the reference period was defined as months 3 and 2 before the date of oral glucocorticoid initiation.

### Clinical indicators

The following putative clinical indicators of adrenal insufficiency were identified in the Danish National Patient Registry (DNPR): hypotension, syncope, cardiovascular collapse, hyponatremia, hypoglycemia, and gastrointestinal symptoms [1]. Among persons with gastrointestinal symptoms, we excluded those with inflammatory bowel disease (IBD) from the analyses. Persons receiving insulin or sulfonylurea treatment were excluded from the hypoglycemia analyses.

Only primary inpatient diagnoses were included to obtain the most accurate date of diagnosis. An exception was syncope; for this indicator, emergency department visits were included in addition to inpatient diagnoses, as medical assessment of this condition usually occurs in the emergency setting. The DNPR has tracked all inpatient stays at Danish public hospitals since 1977, and outpatient clinic and emergency room visits at all public hospitals since 1995. Data recorded in the DNPR include the patient’s civil registration number, dates of admission and discharge or outpatient visit dates, discharge diagnoses for each contact, classified according to the *Eighth Revision* of the *International Classification of Diseases* (ICD-8) until 1994 and the *Tenth Revision* thereafter (ICD-10) [27]. The ICD codes used in this study are provided in S2 Table.

### Statistical analysis

We characterized our study population according to type of last glucocorticoid prescription, duration of treatment (median, IQR), cumulative dose in prednisolone equivalents (median, IQR), average daily dose in prednisolone equivalents, and according to age, sex, morbidity, and concomitant treatment with inhaled, topical or injectable glucocorticoids and glucocorticoids acting on the intestine. The codes for morbidity are provided in S3 Table. Calculations for prednisolone-equivalent cumulative doses are provided in S4 Table.

Based on the entire study population of persons who discontinued oral glucocorticoid treatment, we calculated incidence rates and 2-week prevalence. We estimated incidence rates in the reference period and the five risk periods. Each type of clinical indicator was analysed separately and persons were followed from start of the period of interest until first event, emigration, death or end of the period of interest, whichever came first. We calculated and presented graphically the 2-week prevalence during the period of 12 months before to 7 months after redemption of the last glucocorticoid prescription.

#### Self -controlled case series design

We estimated incidence rate ratios (IRRs) using the self-controlled case series design [22, 28]. This design is a case-only design and each case serves as their own control. Thus, the method inherently accounts for confounding factors that are stable over time (*e*.*g*., sex, ethnicity and genetics). First, we identified cases in the observation period (Fig 2). We analysed each type of clinical indicator as a distinct case variable. In addition, we only considered the first event (*e*.*g*. recurrent events were not included in the analyses). This is the recommended approach by Petersen et al., when recurrent events cannot be assumed independent [22]. Second, we used a conditional fixed-effect Poisson regression to compare incidence rates in the pre-defined risk periods with the incidence rate in the reference period. The follow-up time was not censored at an event. Hence, all time occurring within the observation period (both before and after individuals have experienced the event) was included in the analysis. Nevertheless, individuals were censored at the end of the observation period or death, whichever came first.

In a sub-analysis we stratified on cumulative glucocorticoid dose (< 0.5 g, 0.5–5 g, >5g).

#### Risk factors for adrenal insufficiency

We used Cox proportional hazard regression to identify potential risk factors, such as sex, age (< 30, 30–49, 50–69, and ≥ 70 years of age), treatment duration (< 6 months, 6–12 months, 1–2 years, and > 2 years), cumulative treatment dose (< 0.5 gram (g), 0.5–5 g, > 5 g expressed in prednisolone equivalents), average daily dose (< 5 mg/day, 5–9 mg/day, 10–20 mg/day and > 20 mg/day expressed in prednisolone equivalents), and use of antibiotics. As infections are major precipitating causes of adrenal crisis, we assessed use of antibiotics as a proxy for infection. Antibiotic use was modelled as a time-varying exposure and a person was counted as exposed 30 days after a prescription redemption. We followed our study population from date of cessation until a diagnosis of a clinical indicator (only those indicators showing increased risk during or after withdrawal), death, emigration or end of the observation period, whichever came first. The assumption of proportional hazards was verified graphically.

#### Sensitivity analyses

We conducted several sensitivity analyses to ensure the validity of our results. First, we addressed the concern that if an event increases the probability of death, then the observation periods could be shortened as a direct result of the event. This event-dependent censoring can lead to bias in the self-controlled case series design [22]. Therefore, in a sensitivity analysis we excluded persons who died 60 days after an event. Any major differences in the results of the sensitivity analysis compared to our main results would suggest bias. Second, as the self-controlled case series design can be sensitive to changes in health care utilization, a negative outcome analysis was conducted using erysipelas as outcome. Erysipelas is assumed to be unrelated to both adrenal insufficiency and the condition for which glucocorticoid was prescribed. Third, since alternative routes of glucocorticoid administration (*i*.*e*., injection, inhalation, topical, or glucocorticoids acting on the intestine) also may induce adrenal insufficiency, concomitant use of these types of glucocorticoids could potentially affect our results. To control for this factor, we conducted a sensitivity analysis restricted to persons treated only with oral glucocorticoids.

All statistical analyses were conducted using Stata 14 for Windows.

This study was approved by the Danish Data Protection Agency (Record number: 2016-051-000001, serial number 448). According to Danish legislation, informed consent or approval from an ethical committee is not required for registry based studies.

## Results

In total, we identified 286,680 persons who discontinued long-term (≥ 3 months) oral glucocorticoid treatment (Fig 1). The most frequent type of last redeemed prescription was prednisolone [n = 280,010 (98%)] (Table 1). Median treatment duration was 297 days [interquartile range (IQR): 179–584 days]; median cumulative dose was 3000 mg prednisolone equivalents (IQR: 1,125–6,500 mg), and median average daily dose was 6.8 mg prednisolone equivalents per day (IQR: 4.6–12 mg per day). Median age at cessation was 69 years (IQR: 57–78 years) and 163,077 (57%) were female (Table 1). Inhaled glucocorticoids, topical or glucocorticoids acting on the intestine were used concomitantly by 90,937 persons (32%), and 15,971 persons (5.6%) received glucocorticoid injections (Table 1). Morbidities frequently recorded at any time prior to glucocorticoid cessation included cancer [64,503 (23%)], COPD [63,685 (22%)], asthma [29,573 (10%)], polymyalgia rheumatica/giant cell arteritis [28,220 (9.8%)], and rheumatoid arthritis [21,256 (7.4%)] (Table 1).

**Table 1:** Characteristics of 286,680 oral glucocorticoid users according to sex, age, glucocorticoid type, dose, and morbidity as of the date of cessation.

Characteristics	Number (%)
**Sex**	
Female	163,077 (57)
Male	123,603 (43)
**Age, years**	
0–19	4,188 (1.5)
20–39	21,806 (7.6)
40–59	55,894 (20)
60–79	140,056 (49)
≥80	64,736 (23)
**Type of glucocorticoid use (last prescription)**	
Betamethasone	68 (0.02)
Dexamethasone	215 (0.07)
Methylprednisolone	6,126 (2.1)
Prednisolone	280,010 (98)
Prednisone	0 (0)
Hydrocortisone	261 (0.09)
**Cumulative dose** ^a^	
< 0.5 g	21,114 (7.4)
0.5–5 g	171,566 (60)
5+ g	94,000 (33)
**Average daily dose** ^a^	
< 5 mg /day	86,099 (30)
5–9 mg/day	114,194 (40)
10–20 mg/day	57,970 (20)
20 + mg/day	28,417 (9.9)
**Morbidity**	
Polymyalgia rheumatica/ giant cell arteritis	28,220 (9.8)
Rheumatoid arthritis	21,256 (7.4)
Psoriasis arthritis	1,943 (0.68)
Ankylosing spondylitis	881 (0.31)
Other rheumatological diseases	9,129 (3.2)
Other autoimmune diseases	1,900 (0.66)
Renal diseases	12,568 (4.4)
Cancer	64,503 (23)
Dermatological diseases	1,851 (0.65)
Ulcerative colitis	10,841 (3.8)
Crohn’s disease	6,264 (2.2)
Ashma	29,573 (10)
COPD	63,685 (22)
Multiple sclerosis	1,656 (0.58)
**Concomitant glucocorticoid treatment**^b^	
Locally acting (inhaled, acting on the intestine, or topical)	90,937 (32)
Injections	15,971 (5.6)

^a^ In prednisolone equivalents.

^b^ Defined as prescription redemption throughout the observation period.

In total, 10,963 incident cases of clinical indicators were identified in the observation period (hypotension: 295 cases; syncope: 3,568 cases; cardiovascular collapse: 96 cases; gastrointestinal symptoms: 6,332 cases; hyponatremia: 634 cases; hypoglycemia: 38 cases) (Fig 1). Only 49 persons were coded with treatment-induced adrenal insufficiency during the observation period. Compared to the reference period, the incidence rates of gastrointestinal symptoms, hypotension, cardiovascular collapse, hyponatremia, and hypoglycemia were all elevated in risk period 1 (the withdrawal period) and in risk period 2 (except for hyponatremia). The incidence rate of gastrointestinal symptoms increased to a maximum of 30 per 1000 person-years (P-Y) [95% confidence interval (CI): 28–31 per 1000 P-Y] in risk period 1 (withdrawal period), and remained elevated in risk period 2 with an incidence rate of 24 per 1000 P-Y (95% CI: 22–36 per 1000 P-Y) (Table 2). The incidence rate of hyponatremia was 2.4 per 1000 P-Y (95% CI: 2.0–2.9 per 1000 P-Y) in risk period 1 (Table 2). The incidence rate of hypotension was 0.9 per 1000 P-Y (95% CI: 0.7–1.2 per 1000 P-Y) in risk period 1 and 0.7 per 1000 P-Y (95% CI: 0.5–1.1 per 1000 P-Y) in risk period 2 (Table 2). The incidence rate of hypoglycemia was 0.2 per 1000 P-Y (95% CI: 0.1–0.4 per 1000 P-Y) in risk period 1 and 0.3 per 1000 P-Y (95% CI: 0.1–0.7 per 1000 P-Y) in risk period 2 (Table 2). No diagnoses of cardiovascular collapse occurred in the reference period, but incidence rates in risk periods 1 and 2 were 0.5 per 1000 P-Y (95% CI: 0.3–0.7 per 1000 P-Y) and 0.6 per 1000 P-Y (95% CI: 0.4–0.9 per 1000 P-Y), respectively (Table 2).

**Table 2:** Incidence rates per 1000 person-years with 95% confidence intervals (CI) (n = 286,680).

		Incidence rates per 1000 person years with 95% CIs				
	Reference period	Risk period 0	Risk period 1 (withdrawal period)	Risk period 2	Risk period 3	Risk period 4
Hypotension	0.4 (0.3–0.6)	0.5 (0.5–0.6)	0.9 (0.7–1.2)	0.7 (0.5–1.1)	0.5 (0.3–0.8)	0.4 (0.2–0.7)
Syncope	8.8 (8.0–9.7)	6.1 (5.8–6.3)	8.6 (7.8–9.5)	0.7 (6.6–8.3)	6.7 (5.9–7.6)	5.6 (4.8–6.6)
Cardiovascular collapse	NA	NA	0.5 (0.3–0.7)	0.6 (0.4–0.9)	NA	NA
Hyponatremia	1.7 (1.4–2.1)	0.9 (0.8–1.0)	2.4 (2.0–2.9)	1.4 (1.1–1.9)	1.1 (0.8–1.6)	0.8 (0.5–1.2)
Gastrointestinal symptoms	19 (18–21)	14 (13–14)	30 (28–31)	24 (22–36)	15 (13–16)	14 (12–15)
Hypoglycemia	NA	NA	0.2 (0.1–0.4)	0.3 (0.1–0.7)	NA	NA

NA: Not applicable.

**Fig 3:**
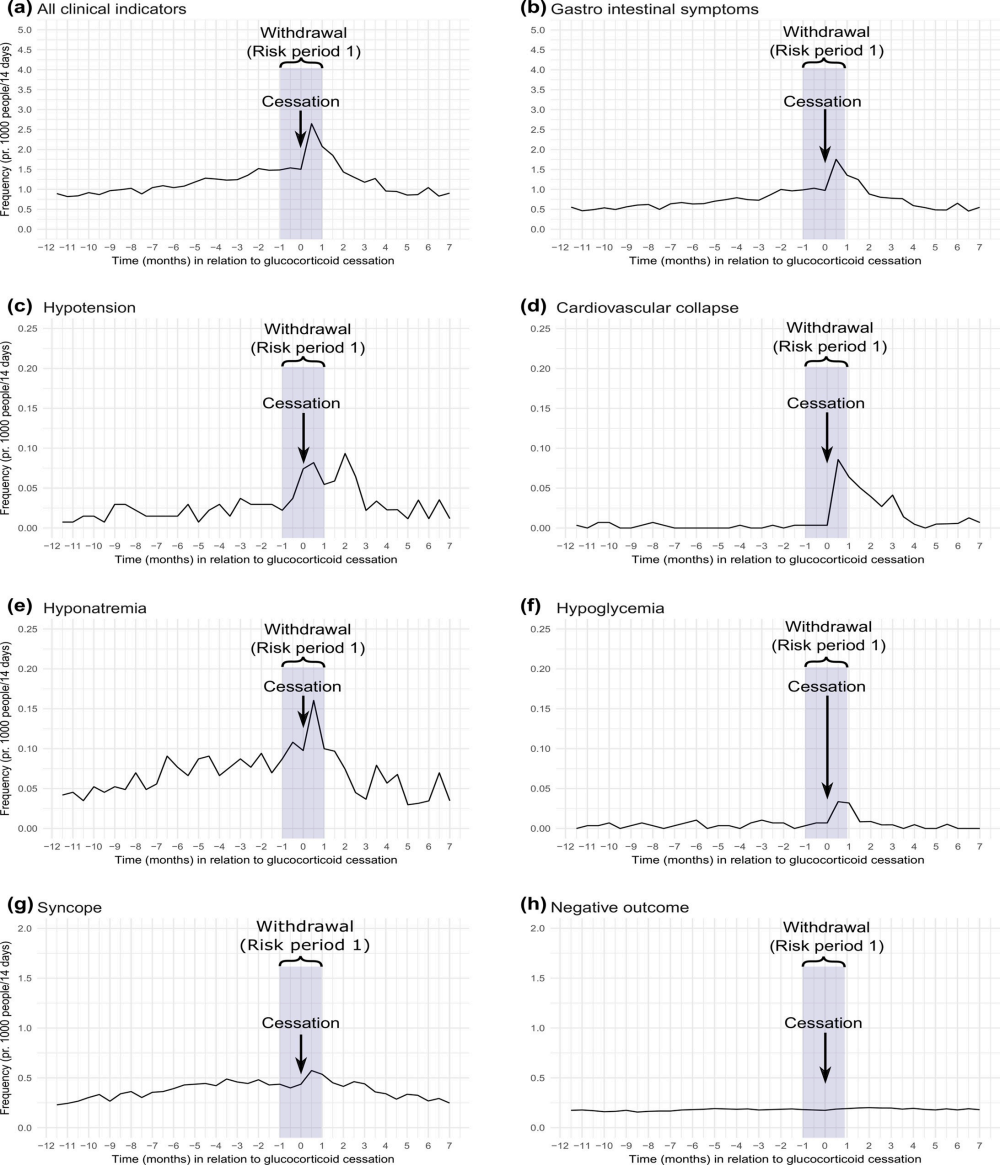
The 2-week prevalence (per 1000 persons) of clinical indicators of adrenal insufficiency and the negative outcome during the period of 12 month before the last glucocorticoid prescription to 7 months after this prescription. (a) All clinical indicators. (b) Gastrointestinal symptoms. (c) Hypotension. (d) Cardiovascular collapse. (e) Hyponatremia. (f) Hypoglycemia. (g) Syncope. (h) Negative outcome.

Fig 3 presents the 2-week prevalence of all clinical indicators of adrenal insufficiency (gastrointestinal symptoms, hypotension, cardiovascular collapse, hyponatremia, hypoglycemia, syncope), and the negative outcome (erysipelas). The prevalence of a composite of all clinical indicators increased to a maximum of 2.6 per 1000 in the 2 weeks after the last glucocorticoid prescription and remained higher than before discontinuation during 1.5 months following the last prescription. Gastrointestinal symptoms had the highest prevalence, with a maximum of 1.8 events per 1000 in the 2 weeks after the last prescription redemption.

The distribution of the total number of admissions per person during risk periods 0–4 is presented in S5 Table.

### Self-controlled case series design

The incidence rates of hypotension, gastrointestinal symptoms, hypoglycemia and hyponatremia were increased in the withdrawal period compared to before treatment started (reference period). The IRRs were 2.5 (95% CI: 1.4–4.3) for hypotension, 1.7 (95% CI: 1.6–1.9) for gastrointestinal symptoms, 2.2 (95% CI: 0.7–7.3) for hypoglycemia, and 1.5 (95% CI: 1.1–2.0) for hyponatremia (Table 3). The risk of hypotension, gastrointestinal symptoms, and hypoglycemia remained elevated, although at a declining rate, during the 7 months of follow up (Table 3). The results of our sensitivity analyses showed that our estimates were robust (S6 and S7 Tables), although the IRRs for hyponatremia attenuated after exclusion of persons with concomitant use of other glucocorticoids (*i*.*e*., those administered by injection, topical, or inhalation, or glucocorticoids acting on the intestine) (S7 Table). No incident cases of cardiovascular collapse were identified in the reference period, so IRRs could not be computed. Our negative outcome (erysipelas) revealed IRRs close to one (Table 3). The results, when stratifying on cumulative glucocorticoid dose, are shown in S8 Table.

**Table 3:** Incidence rate ratios (IRRs) with 95% confidence intervals (CIs) for events by risk period.

	IRR and (95% CI)					
	Syncope	Hypo- natremia	Hypotension	Gastrointestinal symptoms	Hypoglycemia	Negative outcome (erysipelas)
Number of cases	3,568	634	295	6,332	38	1,850
Reference period	1	1	1	1	1	1
Risk period 0	0.8 (0.7–0.9)	0.7 (0.6–1.0)	1.5 (0.9–2.5)	1.0 (0.9–1.1)	0.6 (0.2–2.1)	1.0 (0.8–1.2)
Risk period 1	1.1 (0.9–1.2)	1.5 (1.1–2.0)	2.5 (1.4–4.3)	1.7 (1.6–1.9)	2.2 (0.7–7.3)	1.1 (0.9–1.4)
Risk period 2	1.0 (0.9–1.2)	1.1 (0.7–1.5)	2.3 (1.3–4.3)	2.0 (1.8–2.2)	2.4 (0.6–9.5)	1.0 (0.8–1.3)
Risk period 3	1.0 (0.8–1.2)	0.9 (0.6–1.4)	2.0 (1.0–3.9)	1.5 (1.3–1.7)	NA	1.1 (0.9–1.5)
Risk period 4	0.9 (0.7–1.0)	0.7 (0.4–1.1)	1.7 (0.8–3.6)	1.5 (1.3–1.7)	0.9 (0.1–8.9)	1.1 (0.8–1.4)

### Risk factors

Risk factors for clinical indicators of adrenal insufficiency were use of antibiotics, increasing average daily dose, increasing cumulative dose, and increasing age (Table 4).

**Table 4:** Risk factors for clinical indicators of adrenal insufficiency.

Variable	Hazard ratios and 95% CIs
**Sex**	
Women	1
Men	0.99 (0.92–1.07)
**Age, y**	
< 30	1
30–49	1.24 (0.95–1.63)
50–69	1.80 (1.41–2.32)
≥70	3.00 (2.35–3.82)
**Average daily dose in prednisolone equivalents**	
< 5 mg /day	1
5–9 mg/day	1.13 (1.02–1.25)
10–20 mg/day	1.76 (1.59–1.94)
20 + mg/day	3.28 (2.89–3.73)
**Treatment duration**	
< 6 months	1
6–12 months	1.12 (1.01–1.24)
1–2 years	1.03 (0.92–1.16)
> 2 years	1.21 (1.07–1.36)
**Cumulative dose in prednisolone equivalents**	
< 0.5 g	1
0.5-5g	1.25 (1.10–1.41)
5+ g	2.06 (1.81–2.34)
**Use of antibiotics**^a^	
No	1
Yes	1.83 (1.64–2.05)

^a^ Proxy for infection. The antibiotic prescription had to be redeemed up to 30 days prior to an event to count as a precipitating factor (modelled as a time-varying exposure)

## Discussion

Our main finding were (1) the risks of hypotension, gastrointestinal symptoms, hyponatremia, and hypoglycemia in the glucocorticoid withdrawal period were increased compared to before starting glucocorticoid treatment and remained elevated for hypotension and gastrointestinal symptoms during 7 months of follow up, (2) use of antibiotics, cumulative dose of oral glucocorticoids, average daily dose, and age were associated with increased risk of clinical indicators of adrenal insufficiency during and after withdrawal of glucocorticoid therapy.

To the best of our knowledge, this study is the first to systematically investigate the clinical impact of adrenal insufficiency following oral glucocorticoid withdrawal and it extends anecdotal reports in the literature [17–19]. Prior randomized controlled trials [9, 10] (range: n = 10–42 included persons) and cohort studies [11–15] (range: n = 10–150 persons included) have been unable to establish a time course for adrenal recovery after glucocorticoid cessation. The systematic review by Joseph *et al*. reported a percentage range for biochemical adrenal insufficiency of 27%-69% in patients tested more than 30 days after their last glucocorticoid dose [5]. Our findings suggest that symptomatic adrenal insufficiency peaks up to 3 months after cessation of treatment and remains increased for at least 7 months. Previous studies have debated the association between dose and duration of glucocorticoid treatment and iatrogenic biochemical adrenal insufficiency. In general, high dose and longer treatment duration have been associated with higher adrenal insufficiency risk [4, 5]. Our study confirmed that glucocorticoid dose is a risk factor for clinical adrenal insufficiency. In addition, persons exposed to an infection (potential stressor) were also at increased risk of clinical adrenal insufficiency, which fits well with the underlying pathophysiology of cortisol deficiency [7]. Our study has several strengths. The nationwide population-based design allowed us to assess clinical indicators of adrenal insufficiency in a large heterogeneous population representative of clinical practice. Further, using the self-controlled case series analysis, we were able to take into account patient characteristics that are stable over time, including potential unmeasured or unknown confounders (*i*.*e*. sex, genetics and lifestyle).

Our study also has limitations. First, we used prescription redemption as a proxy for use. Thus, we were unable to assess adherence or to include inpatient hospital medication use. Moreover, we were unable to ascertain the exact timing of the last glucocorticoid dose. It is likely that patients tapered their glucocorticoid therapy for weeks after redemption of their last prescription. To accommodate these inaccuracies, we defined the withdrawal period as a 2-month period encompassing the redemption date of the last prescription. Use of wide risk periods may have biased the IRR estimates toward the null. In addition, we were unable to investigate different tapering schedules.

Second, although the self-controlled case series design accounts for time-independent confounders, it remains sensitive to changes over time in such factors as morbidity or health care utilization. Therefore, confounding by indication or disease severity could affect some of our IRR estimates. For example, gastrointestinal symptoms may be related to IBD relapse. To overcome this, we excluded IBD patients in the assessment of gastrointestinal symptoms. Confounding by use of insulin and sulfonylurea, also were eliminated by exclusion. It is well known that use of other glucocorticoid formulations, such as inhaled and injectable forms and glucocorticoids acting on the intestine, also increases the risk of iatrogenic adrenal insufficiency [29, 30]. In our study, 32% of patients used locally acting glucocorticoids (inhaled, topical, or glucocorticoids acting on the intestine) and 5.6% of patients were treated with glucocorticoid injections in addition to oral glucocorticoids. However, our sensitivity analysis excluding persons with concomitant glucocorticoid treatment did not affect our estimates substantially. We found no association between discontinuation and erysipelas (our negative outcome) indicating that changes in health care utilization were not a major issue.

Third, the self-controlled case series analysis can be sensitive to reverse causation. We did not expect that any of the outcome events would change the decision to prescribe glucocorticoids. However, a change in exposure status from continuous user to discontinued user may be correlated to the terminal phase of illness. Therefore, we were not able to assess death as an outcome. In addition, in the terminal phase, morbidity and frequency of hospitalization often increase, which could potentially lead to incorrect associations. Nevertheless, excluding persons who died 60 days after an event did not alter our results, indicating this was not a major issue in our study.

Fourth, our use of hospital registry data to assess outcomes raises the possibility of misclassification. Hyponatremia and cardiovascular collapse diagnoses have been validated and the positive predictive values are > 90%, however, other outcomes used in our study lack validation [27]. Still, misclassification of outcomes is unlikely to vary systematically according to reference and risk periods. Thus, it could not explain any associations found in the self-controlled case series analysis. Another concern is that low data completeness could lead to underestimation of our incidence rates and 2-week prevalence. Furthermore, the defined clinical indicators are not specific for adrenal insufficiency. In addition, we were only able to assess outcomes that led to a hospital contact. Hence, less severe indicators, such as fatigue and muscle weakness, were not captured. We also lacked access to biochemical data, hence cortisol measurements could not be assessed. Finally, only 49 persons in our study had a recorded diagnosis of treatment-induced adrenal insufficiency. This small number most likely reflects lack of awareness and failure to diagnose iatrogenic adrenal insufficiency, considering that discontinuation of glucocorticoids may induce biochemical adrenal insufficiency in 50% of patients [4].

In conclusion, we found that oral glucocorticoid withdrawal was associated with observable clinical outcomes attributable to adrenal insufficiency, although the incidence rates were low. Our study underscores the importance for future research to establish evidence-based clinical guidance on procedures for patients’ withdrawal from glucocorticoid use, as well as guidance for identifying patients in need of biochemical testing.

## Supporting information

S1 Table Anatomical Therapeutic Classification (ATC) codes, Nordic article numbers, and procedure codes for medications. (PDF)

S2 Table *Tenth Revision of the International Classification of Diseases* (ICD-10) codes for outcome events. (PDF)

S3 Table Morbidity. *Eighth Revision of the International Classification of Diseases* (ICD-8) until 1994 and the *Tenth Revision* (IDC-10) codes.
(PDF)

S4 Table Equivalency table presenting systemic glucocorticoids and corresponding prednisolone conversion factors. Cumulative dose calculation: The cumulative dose was calculated by multiplying the number of pills, dose per pill, and prednisolone conversion factor for each prescription and then adding them up across all prescriptions.
(PDF)

S5 Table Distribution of total number of hospital admissions per person during risk periods 0–4. (PDF)

S6 Table Sensitivity analysis excluding persons who died within 60 days after an event. Incidence rate ratios (IRRs) and 95% confidence intervals (CIs) for events by risk period.
(PDF)

S7 Table Sensitivity analysis excluding persons with concomitant use of injectable or locally administrated glucocorticoids. Incidence rate ratios (IRRs) and 95% confidence intervals (CIs) for events by risk period.
(PDF)

S8 Table Incidence rate ratios (IRRs) with 95% confidence intervals (CIs) for events by risk period. Sub-analysis stratified on cumulative glucocorticoid dose.
(PDF)
