# Supplementary material for: Clinical indicators of adrenal insufficiency following discontinuation of oral glucocorticoid therapy: A Danish population-based self-controlled case series analysis
Source: PLoS One. 2019 Feb 19;14(2):e0212259. doi: 10.1371/journal.pone.0212259 (PMC6380588; doi:10.1371/journal.pone.0212259)
Supplement: S1 Table — (PDF) [file pone.0212259.s001.pdf]

Drug	ATC code	Nordic article number	Procedure codes
**Oral glucocorticoids**			
Betamethasone	H02AB01	499590	
Dexamethasone	H02AB02	039413; 126955; 188988; 113331; 190108; 190132; 374319; 418122; 579043; 591445	
Methylprednisolone	H02AB04	046557; 050487; 072601; 109119; 111245; 112671; 159103; 174284; 450619; 499772; 500074; 509125; 509133; 536422	
Prednisolone	H02AB06	042448; 164118; 164130; 164141; 164153; 164164; 164175; 168548; 184382; 398747; 425905; 502076; 507660; 516005; 516013; 516021; 516039; 521930; 521948; 521955; 530899: 564513; 743542; 743559; 743567;748756	
Prednisone	H02AB07		
Hydrocortisone	H02AB09	049319; 141569; 155579; 393735; 424199; 445320; 487361; 490667; 490667; 503581; 746628; 746636	
**Glucocorticoids by injection**			
Betamethasone	H02AB01	006595; 006634; 013802; 013815; 013824; 013835; 034731; 038661; 042812; 058396; 123372; 131796; 143773; 145143; 177360; 181595; 192914; 192922; 194713; 385214; 399765; 413438; 473824; 477631; 483156; 488471; 498048, 504332; 523266; 556860	
Dexamethasone	H02AB02	053066; 057984; 421131; 570853; 570861	
Methylprednisolone	H02AB04	042093; 047663; 067283; 130683; 134940; 134965; 141044; 143339; 143347; 153928; 161075; 161637; 165613; 171016;180299; 189506; 189522; 195389; 390762; 397856; 420151; 434274; 453162; 465187; 488496; 489011; 530391; 549923; 560425; 563956; 583158; 590659	
Prednisolone	H02AB06	189811	
Triamcinolone	H02AB08		
**Locally-acting glucocorticoids**			
Beclomethasone (inhaled)	R03BA01; R03AK08		
Budesonide (inhaled)	R03BA02; R03AK07		
Flunisolide (inhaled)	R03BA03		
Fluticasone (inhaled)	R03BA05; R03AK06; R03AK10; R03AK11		
Mometasone (inhaled)	R03BA07		
Ciclesonide	R03BA08		
Prednisolone (acting on the intestine)	A07EA01		
Hydrocortisone (acting on the intestine)	A07EA02		
Budesonide (acting on the intestine)	A07EA06		
Various glucocorticoids for hemorrhoids	C05AA		
Prednisolone (suppositories)	H02AB06	685546	
Glucocorticoids for skin conditions	D07		
**Treatments for IBD besides oral glucocorticoids**			
Hydrocortisone (acting on the intestine)	A07EA02		
Budesonide	A07EA06		
Azathioprin	L04AX01		
Methotrexate	L01BA01, L04AX03		BWHA115
Mesalazin	A07EC02		
Sulfazalazine	A07EC01		
Infliximab	L04AB02		BOHJ18A1
Adalinumab	L04AB04		BOHJ18A3
Vedolizumab	L04AA33		BOHJ19H4
Ustekinumnab	L04AC05		BOHJ18B3
Golinumab	L04AB06		BOHJ18A4

**Other medications**	
Insulin	A10A
Sulfonylureas	A10BB
**Antibiotics**	J01
