# Supplementary material for: Clinical indicators of adrenal insufficiency following discontinuation of oral glucocorticoid therapy: A Danish population-based self-controlled case series analysis
Source: PLoS One. 2019 Feb 19;14(2):e0212259. doi: 10.1371/journal.pone.0212259 (PMC6380588; doi:10.1371/journal.pone.0212259)
Supplement: S2 Table — (PDF) [file pone.0212259.s002.pdf]

**Diagnoses**	**ICD-10 codes**
Syncope	R55
Hyponatraemia	E871
Hypotension	I95
Cardiovascular collapse	
Cardiogenic shock or	R570
Hypovolemic shock	R571
Gastrointestinal symptoms	
Nausea/vomiting	R11
Abdominal pain	R10
Diarrhea	K529B
Obstipation	K590
Hypoglycaemia	E15 E162
Treatment-induced adrenal insufficiency	E273
Erysipelas (negative outcome)	A46
