# Supplementary material for: Clinical indicators of adrenal insufficiency following discontinuation of oral glucocorticoid therapy: A Danish population-based self-controlled case series analysis
Source: PLoS One. 2019 Feb 19;14(2):e0212259. doi: 10.1371/journal.pone.0212259 (PMC6380588; doi:10.1371/journal.pone.0212259)
Supplement: S3 Table — Eighth Revision of the International Classification of Diseases (ICD-8) until 1994 and the Tenth Revision (IDC-10) codes. (PDF) [file pone.0212259.s003.pdf]

Disease	ICD-10	ICD-8
**Pulmonary diseases**		
Asthma	J45, J46	493
Chronic obstructive pulmonary disease	J41, J42, J43, J44	491, 492
**Rheumatic diseases**		
Polymyalgia rheumatica/ Giant cell arthritis	M315, M316, M35.3	446.30, 446.31, 446.39
Rheumatoid arthritis	M05, M06	712.19, 712.29, 712.39, 712.59
Psoriasis arthritis	M07.0-M07.3	696.09
Ankylosing spondylitis	M45	712.49
Other rheumatic diseases	L94.0, L94.1 (Sclerodermia) M35.1 (mixed connective disease) M34.0-9 (LE), M32, G73.7C, N08.5A, N16.4B (SLE), M33 (polymyositis/dermatomyositis). M35.0, G73.7A (Sjögren's syndrome) M30.0 (Polyarteritis nodosa) M31.3 (Wegener´s granulomatosis) D69.0B, M31.0B (Schonlein henochs purpura) I77.6, DL95 (Vasculitis/arteritis)	734.00, 734.02, 734.03, 734.04 734.08, 734.09 (Sclerodermia) 695.49 (LE), 734.19 (SLE) 716.09, 716.19 (polymyositis/dermatomyositis), 734.90 (Sjögren's syndrome) 446.29 (Wegener´s granulomatosis) 287.09 (Schonlein henochs purpura) 446.09 (Vasculitis/arteritis)
**Gastrointestinal diseases**		
Crohn's disease	K500-509	563.01, 563.02, 563.09
Ulcerative colitis	K510-519	563.19
Unclassified IBD	K519, DK529	
**Cancer**	C00-97	140-209
**Dermatological diseases** Pemphigus / pemphigoid dermatitis herpetiformis Bullous disorders	L10.0, L10.2, L10.4, L12.0, L13.0, L00, L51.2, L11, L13,14	694 , 693.00, 693.08, 693.09, 684.00
**Renal diseases**	N00, N01, N03, N04, N05 N06, N07, N08, N11, N14, N15, N16, N18, N19, N26, N27, I12. I13, I15.0, I15.1, E10.2, E11.2, E14.2, Q61.1-Q61.4	249.02, 250.02, 403, 404, 580-584, 590.09, 593.20, 753.10-753.19
**Other autoimmune diseases**	D59.0<br>D59.1 (autoimmune hemolytic anemia)<br>D69.3 (Idiopathic thrombocytopenic purpura)<br>K75.4 (autoimmune hepatitis)	283.90<br>283.91 (autoimmune hemolytic anemia)<br>287.10 (Idiopathic thrombocytopenic purpura)<br>571.93 (autoimmune hepatitis)
**Neurological diseases**		
Multiple sclerosis	G35	340
**Adrenal insufficiency**	E230, E240, E271, E272, E274, E893	253, 25510, 25511
