# Supplementary material for: Clinical indicators of adrenal insufficiency following discontinuation of oral glucocorticoid therapy: A Danish population-based self-controlled case series analysis
Source: PLoS One. 2019 Feb 19;14(2):e0212259. doi: 10.1371/journal.pone.0212259 (PMC6380588; doi:10.1371/journal.pone.0212259)
Supplement: S4 Table — Cumulative dose calculation: The cumulative dose was calculated by multiplying the number of pills, dose per pill, and prednisolone conversion factor for each prescription and then adding them up across all prescriptions. (PDF) [file pone.0212259.s004.pdf]

	**Equivalent glucocorticoid dose**	**Prednisolone conversion factor**
Cortisone	25	0.2
Cortisol	20	0.25
Methylprednisolone	4	1.25
Prednisolone	5	1
Prednisone	5	1
Triamcinolone	4	1.25
Dexamethasone	0.75	6.67
Betamethasone	0.6	8.33

Cumulative dose calculation:

The cumulative dose was calculated by multiplying the number of pills, dose per pill, and prednisolone conversion factor for each prescription and then adding them up across all prescriptions.
