# Supplementary material for: Clinical indicators of adrenal insufficiency following discontinuation of oral glucocorticoid therapy: A Danish population-based self-controlled case series analysis
Source: PLoS One. 2019 Feb 19;14(2):e0212259. doi: 10.1371/journal.pone.0212259 (PMC6380588; doi:10.1371/journal.pone.0212259)
Supplement: S5 Table — (PDF) [file pone.0212259.s005.pdf]

**Number of admissions in risk period 0-4 per person**	**Number (%)**
Persons with any admission	9,058 (100)
Persons with only 1 admission in total	7,517 (83)
Persons with 2 admissions in total	1,158 (13)
Persons with 3 admissions in total	260 (2.9)
Persons with 4 admissions in total	68 (0.75)
Persons with 5 admissions in total	30 (0.33)
Persons with 6 admissions in total	14 (0.15)
Persons with ≥ 7 admissions in total	11 (0.12)
