# Supplementary material for: Clinical indicators of adrenal insufficiency following discontinuation of oral glucocorticoid therapy: A Danish population-based self-controlled case series analysis
Source: PLoS One. 2019 Feb 19;14(2):e0212259. doi: 10.1371/journal.pone.0212259 (PMC6380588; doi:10.1371/journal.pone.0212259)
Supplement: S6 Table — Incidence rate ratios (IRRs) and 95% confidence intervals (CIs) for events by risk period. (PDF) [file pone.0212259.s006.pdf]

	IRR and (95% CI)				
	**Syncope**	**Hypo-natremia**	**Hypotension**	**Gastrointestinal symptoms**	**Hypoglycemia**
Number of cases	3,527	629	292	6,220	37
Reference period	1	1	1	1	1
Risk period 0	0.8 (0.7 - 0.9)	0.7 (0.6 - 1.0)	1.5 (0.9 - 2.5)	1.0 (0.9 - 1.1)	0.7 (0.2 - 2.2)
Risk period 1	1.0 (0.9 - 1.2)	1.4 (1.1 - 2.0)	2.3 (1.3 - 4.1)	1.6 (1.5 - 1.8)	2.2 (0.7 - 7.3)
Risk period 2	1.0 (0.8 - 1.1)	1.1 (0.7 - 1.5)	2.2 (1.2 - 4.1)	1.8 (1.6 - 2.0)	1.9 (0.5 - 7.9)
Risk period 3	1.0 (0.8 - 1.1)	0.9 (0.6 - 1.4)	1.9 (1.0 - 3.8)	1.4 (1.3 - 1.6)	NA
Risk period 4	0.8 (0.7 - 1.0)	0.7 (0.4 - 1.1)	1.7 (0.8 - 3.5)	1.4 (1.3 - 1.7)	0.9 (0.1 - 8.5)
