# Supplementary material for: Clinical indicators of adrenal insufficiency following discontinuation of oral glucocorticoid therapy: A Danish population-based self-controlled case series analysis
Source: PLoS One. 2019 Feb 19;14(2):e0212259. doi: 10.1371/journal.pone.0212259 (PMC6380588; doi:10.1371/journal.pone.0212259)
Supplement: S7 Table — Incidence rate ratios (IRRs) and 95% confidence intervals (CIs) for events by risk period. (PDF) [file pone.0212259.s007.pdf]

	IRR and (95% CI)				
	**Syncope**	**Hypo-natremia**	**Hypotension**	**Gastro intestinal symptoms**	**Hypoglycemia**
Number of cases	2,367	376	192	3,904	28
Reference period	1	1	1	1	1
Risk period 0	0.8 (0.7 - 0.9)	0.7 (0.5 - 1.0)	1.2 (0.7 - 2.1)	1.0 (0.9 - 1.1)	0.8 (0.2 - 3.1)
Risk period 1	1.0 (0.8 - 1.2)	1.1 (0.7 - 1.6)	2.2 (1.2 - 4.1)	1.6 (1.4 - 1.8)	1.9 (0.5 - 8.0)
Risk period 2	0.9 (0.8 - 1.1)	0.7 (0.4 - 1.2)	1.2 (0.6 - 2.5)	1.7 (1.5 - 2.0)	3.0 (0.6 - 15)
Risk period 3	1.0 (0.8 - 1.2)	0.7 (0.4 - 1.2)	1.3 (0.6 - 2.9)	1.2 (1.0 - 1.5)	NA
Risk period 4	0.8 (0.6 - 1.0)	0.6 (0.3 - 1.1)	1.6 (0.7 - 3.7)	1.3 (1.1 - 1.6)	1.3 (0.1 - 14)
