# Supplementary material for: Clinical indicators of adrenal insufficiency following discontinuation of oral glucocorticoid therapy: A Danish population-based self-controlled case series analysis
Source: PLoS One. 2019 Feb 19;14(2):e0212259. doi: 10.1371/journal.pone.0212259 (PMC6380588; doi:10.1371/journal.pone.0212259)
Supplement: S8 Table — Sub-analysis stratified on cumulative glucocorticoid dose. (PDF) [file pone.0212259.s008.pdf]

	IRR and 95% CI				
	Syncope	Hypo-natremia	Hypotension	Gastrointestinal symptoms	Hypoglycemia
Reference period	1	1	1	1	1
**Cumulative dose < 0.5 g**					
Cases	87	16	6	179	0
Risk period 0	1.1 (0.5 – 3.5)	2.0 (0.4 – 9.8)	NA	0.7 (0.4 – 1.4)	NA
Risk period 1	1.3 (0.5 – 3.1)	1.1 (0.1 - 11)	NA	0.8 (0.4 – 1.6)	NA
Risk period 2	0.9 (0.4 – 1.8)	0.3 (0.03 – 3.2)	NA	1.2 (0.8 – 1.9)	NA
Risk period 3	1.1 (0.6 – 3.5)	2.5 (0.6 – 9.6)	NA	1.2 (0.7 – 1.9)	NA
Risk period 4	1.7 (0.8 – 3.5)	0.5 (0.04 – 4.8)	NA	1.8 (1.1 – 2.9)	NA
**Cumulative dose 0.5-5 g**					
Cases	1,432	263	117	2,501	15
Risk period 0	0.8 (0.7 – 0.9)	0.8 (0.6 – 1.2)	1.2 (0.6 – 2.4)	1.1 (1.0 – 1.3)	0.4 (0.1 – 1.8)
Risk period 1	1.0 (0.9 – 1.3)	1.3 (0.9 – 2.0)	2.2 (1.1 – 4.4)	1.7 (1.5 – 2.0)	0.7 (0.1 – 4.3)
Risk period 2	1.0 (0.8 – 1.2)	0.9 (0.6 – 1.5)	2.6 (1.3 – 5.4)	1.9 (1.7 – 2.3)	1.1 (0.2 – 6.7)
Risk period 3	1.0 (0.8 – 1.2)	0.7 (0.4 – 1.2)	2.0 (0.9 – 4.4)	1.5 (1.3 – 1.8)	NA
Risk period 4	0.8 (0.6 – 1.0)	0.6 (0.3 – 1.1)	0.9 (0.3 – 2.7)	1.5 (1.2 – 1.8)	NA
**Cumulative dose > 5 g**					
Cases	2,049	355	172	3,652	23
Risk period 0	0.8 (0.7 – 1.0)	0.7 (0.5 – 1.0)	1.8 (0.8 – 4.2)	1.0 (0.9 – 1.1)	1.4 (0.2 - 12)
Risk period 1	1.1 (0.9 – 1.3)	1.8 (1.1 – 2.8)	3.2 (1.3 – 8.2)	1.8 (1.6 – 2.1)	6.7 (0.8 - 56)
Risk period 2	1.1 (0.8 – 1.4)	1.4 (0.8 – 2.5)	1.4 (0.4 – 5.1)	2.1 (1.8 – 2.5)	7.0 (0.7 - 71)
Risk period 3	1.0 (0.7 – 1.4)	1.2 (0.6 – 2.5)	2.6 (0.8 – 8.7)	1.5 (1.1 – 1.9)	NA
Risk period 4	0.8 (0.6 – 1.2)	0.7 (0.3 – 1.9)	4.2 (1.3 - 14)	1.4 (1.1 – 1.9)	7.0 (0.4 - 129)
